# Polyphenols and Flavonoids Composition, Anti-Inflammatory and Antioxidant Properties of Andean *Baccharis macrantha* Extracts

**DOI:** 10.3390/plants11121555

**Published:** 2022-06-12

**Authors:** Santiago Rosero, Freddy Del Pozo, Walter Simbaña, Mario Álvarez, María Fernanda Quinteros, Wilman Carrillo, Dayana Morales

**Affiliations:** 1 Department of Research, Laboratory of Functional Foods, Department of Science and Engineering in Food and Biotechnology, Campus Huachi, Technical University of Ambato, Av. Los Chasquis y Río Payamino, Ambato 1801334, Ecuador; sa.rosero@uta.edu.ec (S.R.); fg.delpozo@uta.edu.ec (F.D.P.); wa.simbana@uta.edu.ec (W.S.); mf.alvarez@uta.edu.ec (M.Á.); 2 Instituto Nacional de Biodiversidad (INABIO), Pje. Rumipamba 341 y Av. De los Shyris, Quito170506, Ecuador; 3 Departamento de Investigación, Universidad Estatal de Bolívar, Guaranda-Bolívar 020102, Ecuador; mquinteros@ueb.edu.ec; 4 Departamento de Ingeniería Rural y Agroalimentaria, Universidad Politécnica de Valencia, 46022 Valencia, Spain

**Keywords:** *Baccharis macrantha*, extracts, polyphenols, flavonoids, antioxidant activity, anti-inflammatory activity, TBARS

## Abstract

This study examined the leaves of *Baccharis macrantha* to obtain extracts of *Baccharis macrantha* (EBM) and to determine the total flavonoid content (TFC) and the total polyphenol content (TPC). The main objective of this work was to quantify TPC and TFC of extracts of *B. macrantha* from Ecuador and evaluate its antioxidant and anti-inflammatory activities and inhibition of lipid peroxidation. The extraction method was optimized with solvents, ethanol, and methanol, at temperatures of 30–60 °C and extraction times of 5–20 min. The optimal TFC extraction conditions were at EtOH25% at 50 °C for 10 min. The optimal TPC extraction conditions were at EtOH50% at 50 °C for 10 min. EBM was characterized by TLC and HPLC with three standards: gallic acid, catechin, and quercetin. EBM-EtOH25% and EBM-EtOH50% obtained at 50 °C for 10 min were used to identify quercetin and evaluate biologicals activities. Quercetin was detected in EBM (EtOH25% and EtOH50%). EBM anti-inflammatory activity was evaluated with the red blood cell stabilization (RBC) method. The RBC model showed values of 49.72% of protection lysis RBC to EBM-EtOH25% and 50.71% of protection lysis RBC to EBM-EtOH50%. The EBM in vitro inhibition of lipid peroxidation showed a protection of 77.00% (EtOH25%) and 73.11% (EtOH50%) when the TBARs method was used. EBM-EtOH25% and EtOH50% showed high antioxidant activity. EBM-EtOH25% presented values of ABTS (1172 µmol TE/g EBM), DPPH (836 µmol TE/g, EBM), and FRAP (85.70 µmol TE/g, EBM).

## 1. Introduction

Plants have been used as medicines for more than 5000 years by mankind to treat different diseases. Ethnobotanical and ethnopharmacological science define medicinal plants as plant species used in traditional medicine that contain beneficial elements in the cure and treatment of diseases in humans and/or animals. Many medicinal plants are used in traditional medicine systems, such as the Indian Ayurvedic medicine system, traditional Chinese medicine, and the West Indian and Latin American folk medicine system. A large part of the population in developing countries continues to use ancestral medicines based on medicinal plant extracts [1,2,3,4,5].

The South America tropical Andes constitute the most species-rich biodiversity hotspot worldwide with around 15% of the world plant species, on just 1% of the world’s land area [6]. 17,548 vascular plant species are present in Ecuador, many of which play a major role in the development of Andean cultures. 5172 useful species have been reported by Ecuador, of which 60% are medicinal plants, 55% are a source of materials, 30% are edible, and 20% have social uses. With regard to the geographical location of the uses, 42% come from the eastern lowlands, 47% from the Andes, and 12% from the lowlands of the Coast and the Galapagos Islands [7,8]. Native Ecuador flora is used for ancestral medicinal purposes, where the genus *Baccharis* is included.

*Baccharis* is a dioecious, neotropical genus and the most diverse in the Asteraceae family [9,10,11]. It has between 354 to 500 species of shrubs, perennial herbaceous, sub-shrubs, aromatic, and native to the American continent [12,13,14]. The regions of the genus with the highest taxonomic richness are the tropical Andes and southeastern Brazil [15]. These plants have revealed medicinal properties used by the traditional medicine [16,17,18]. Over 150 compounds have been identified and isolated in the genus [14], including diterpenes [19], triterpenes [20,21,22], and flavonoids [23]. Different extracts of *Baccharis* plants have been used to evaluate the biological properties of their phytocomposition. Aqueous extracts from *B. articulata*, *B. crispa*, and *B. trimera* with anti-inflammatory properties have been described by Gené et al. [24,25]. Aqueous, methanol, and dichloromethane extracts from *B. grisebachii*, *B. incarum*, and *B. latifolia* were studied with activity on the respiratory burst and the inducible heat shock protein of 72 kDa (hsp72) synthesis. Dichloromethane extracts of *B. grisebachii* showed the higher activity in the assays [26]. Oliveira et al. [27] reported extracts from *B. articulata* with antioxidant activity. They identified the phenolic compound (4′-O-β-D-glucopyranosyl-3′,5′-dimethoxybenzyl-caffeate) with antioxidant activity similar to the Trolox standard used as positive control in antioxidants assays. Oliveira et al. [28] reported antioxidant activity of aqueous, dichloromethane, and ethanol extracts from *B. spicata*, *B. trimera*, and *B. usterii*, using the FRAP and TBARS methods.

Ecuador is home of 46 native *Baccharis* species, of which 11 are endemic. Traditionally, these species have been used for food, medicine, social, material, beekeeping, or fuel. One of the native species is *B. macrantha* Kunth, locally known as tigra, chilca, or pince; this species grows in the provinces of Azuay, Bolívar, Carchi, Chimborazo, Cotopaxi, Imbabura, Loja, Morona Santiago, Napo, Pichincha, Tungurahua [29].

The most studied characteristic of phenolic compounds is the ability to protect against oxidative damage in the human body. Protection against peroxidation of lipoproteins could prevent heart diseases. Protection against oxidative DNA damage could protect against certain types of cancer [30,31]. Epidemiological studies suggest that there is a correlation between the high consumption of phenolic compounds in the diet and the reduction of cardiovascular disease risks [32,33]. The main antioxidant activity associated with polyphenols is the ability to scavenge free radicals. Antioxidant and anti-inflammatory activities of medicinal plants are related to a high content of polyphenols and flavonoids in different parts of plants (fruits, seeds, leaves, and roots) [34,35]. Modern science is interested in verifying these properties by analyzing the medicinal plants for the presence of valuable bioactive compounds, including antioxidants compounds and the resulting interesting potential health properties. About 25% of current pharmaceuticals are derived directly or indirectly from plants, highlighting the strong foundation of plant-derived medicines [36].

*B. macrantha* is used in Ecuador as an ancestral medicinal plant with anti-inflammatory activity. However, there are no studies in the literature about its phytochemical composition and the evaluation of its antioxidant and anti-inflammatory activity. A detailed description of the mineral composition when *B. macrantha* grows in volcanic areas is inexistent. This study quantifies secondary metabolites (polyphenols and flavonoids) of extracts of *B. macrantha* and evaluates the effects on the inhibition of lipid peroxidation, antioxidant, and anti-inflammatory activities and determines its mineral composition.

## 2. Materials and Methods

### 2.1. Chemicals

Folin-Ciocalteu reactive, reference standard quercetin dihydrate (purity 99% *w*/*w*), catechin (98% *w*/*w*), quercetin (purity 99% *w*/*w*), gallic acid, and butylhydroxytoluene (BHT) standards were obtained from Sigma Chemical Co. (St Louis, MO, USA). Ethanol and methanol of grade HPLC were used.

### 2.2. Plant Material

**Figure 1:**
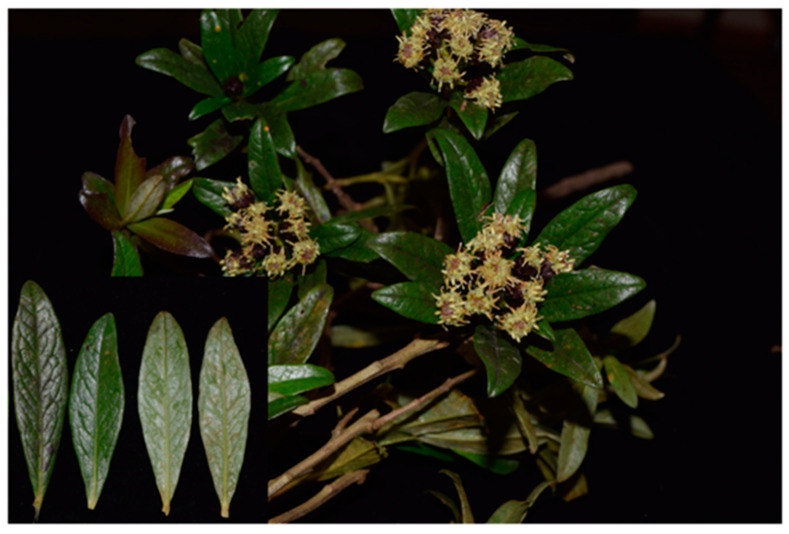
The branches of *Baccharis macrantha* with the flowering, the leaves with a shiny surface, and the underside with creamy-white wax (lower left side).

*Baccharis macrantha* Kunth is an aromatic shrub of 7.8 m tall with a main trunk diameter of 11 to 32 cm. The leaves are simple, alternate, elliptic-oblong, oblong-ovate, oblanceolate-elliptic, sublinear, or linear, coriaceous; shiny top and back with creamy-white wax. Inflorescence is terminal and axillary. The peduncle measures more than 8–10 mm in diameter. The involucre has 30–60 filariae, and 6–7-serrated are generally purple (Figure 1).

Between May and October 2018, the leaves of 10 adult *B. macrantha* individuals were collected in the Andean Forest of Cerro Teligote with GPS coordinates (01°21′58.7″ S) and UTM (78°33′41.8″ W) at 3324 m of altitude, in the community of Teligote, about 6.5 km southwest of the San Pedro de Pelileo canton, Tungurahua Province, central highlands of Ecuador. The collected plant material was kept inside a Ziploc plastic bag and then transported to the FCIAB-UTA laboratories. The *B. macrantha* plants were identified by the National Herbarium of Ecuador (QNCE). They were compared with stored *B. macrantha* plants from QNCE, the herbarium photographs, and the specific taxonomic literature of the *Baccharis* species. A part of the samples was stored in the Misael Acosta Solís Herbarium (AMAS) of the Technical University of Ambato. Registry number 1011(AMAS0000037).

### 2.3. Optimization of Polyphenols and Flavonoids Extraction by the Solid-Liquid Phase Extraction

The extraction process (solid-liquid phase extraction) is an important step to quantify phenolic components. To optimize the parameters of the extraction of polyphenols and flavonoids from *Baccharis macrantha* leaves the effect of three independent variables (extraction solvent, extraction temperature and extraction time) was evaluated in two responses named: total polyphenols content (TPC) and total flavonoids content (TFC). The extraction solvent variable was examined in nine levels [water, ethanol (25, 50, 75 and 96%) and methanol (25, 50, 75 and 98%)]. The extraction temperature variable was evaluated in two levels (50 °C and 60 °C) and the extraction time variable was evaluated in three levels (5, 10 y 20 min).

*Extraction solvent:* Two kinds of solvent systems were tested for their ability to extract secondary metabolites, polyphenols, and flavonoids. Unitary solvent systems (ethanol, methanol, and water) and binary solvent systems (hydroethanolic and hydro methanolic at concentrations of 25, 50, and, 75% *w*/*v*) were used.

The test began with 0.1 g of powder plant material being extracted with 1 mL of each solvent in an ultrasound bath (Branson 2800 Sonicator, Rochester, NY, USA) for 5 min at 50 °C. The mixtures were centrifuged at 12,000× *g* at 10 °C for 10 min. The supernatant was concentrated in an oven at 40 °C and increased to 5 mL with distilled deionized water. The extracts obtained were used to determine TPC and TFC contents.

Extraction temperature: Once the most effective solvent was selected, extractions were performed at different temperatures (30, 40, 50, and, 60 °C), following the same procedure described above.

Extraction time: Finally, with the best conditions for solvent and temperature, three different periods were tested (5, 10, and, 20 min) in the extraction process.

Once the best conditions related to the solvent, temperature, and time were defined, the vegetal material was re-extracted four more times under the same conditions. Then, the extraction process was carried out with additional vegetal material maintaining the same solid-solvent ratio (1:10, *w*/*v*). Extraction was carried out five times under the same conditions. All assays were made in triplicate (*n* = 3).

### 2.4. Total Polyphenol Content (TPC)

TPC was determined with the Folin-Ciocalteu method according to the description by Vasco et al. [37]. In a 10 mL flask, 50 µL of EBM and 100 µL of Folin-Ciocalteu reagent were mixed. The solution was stirred for 3 min before adding 2 mL of NaCO_3_ (75 g/L). The volume was increased to 5 mL with distilled water. The solution was allowed to stand for 2 h at room temperature and in dark conditions. A blank was prepared in which the EBM was replaced with an equal volume of distilled water. The absorbance of the blue-colored complex was measured at 750 nm in a spectrophotometer (Evolution 201, Thermo Scientific, Waltham, MA, USA). TPC concentration was calculated with a calibration curve over the range of 50–200 ppm of gallic acid. The curve calibration obtained was (y = 0.0022x + 0.0268; R^2^ = 0.9979). The results obtained were expressed as mg of gallic acid equivalents (GAE)/g of EBM, dry weight (DW). All assays were made in triplicate (*n* = 3).

### 2.5. Total Flavonoid Content (TFC)

TFC was determined using the colorimetric method described by Sakanaka et al. [38]. 1.25 mL of water + 0.25 mL of EBM + 0.075 mL of (5% NaNO_3_) leaving the mixture for 6 min. Then, 0.15 mL of AlCl_3_ solution was added. The solution was standing up for 5 min. After, 0.5 mL of 1 M NaOH was added. The volume was increased to 5 mL with distilled water. The solution was mixed, and then the rosacea complex was measured at 510 nm. The calibration curve of the (+) catechin (CT) standard was made in the range of (5–100 ppm). The curve obtained was (y = 0.0289x + 0.0198; R^2^ = 0.9949). The results obtained were expressed as mg of catechin equivalents (mg CT/g of EBM, dry weight DW). All assays were carried out in triplicate (*n* = 3).

### 2.6. Thin-Layer Chromatography (TLC)

TLC analysis was made on a precoated silica gel 60 F254 TLC plate (Merck, Darmstadt, Germany) according to the method described by Khatoon et al. [39] with some modifications. Aliquots (6 µL) of ethanolic and methanolic EBM (15 mg/mL) and standards solutions (5 mg/mL of gallic acid, quercetin and (+) catechin) previously filtered (0.45 µm nylon membrane filters) were loaded on the activated plates as bands. The plates were developed in vertical glass chambers previously saturated with the mobile phase (toluene: ethyl acetate: formic acid) (40:40:2). After development, plates were dried at room temperature for 5 min and phenolic compounds were detected under UV light at 366 nm.

### 2.7. Reverse Phase-Ultra High-Performance Liquid Chromatography (RP-UHPLC)

UHPLC analysis was performed using an UHPLC on Agilent 1200 infinity series UHPLC System (Agilent Technologies, Waldron, Germany) with an XDB-C18 (Agilent Zorbax Eclipse 4.6 mm × 25 cm × 5.0 µm of particle size) column and water-methanol as mobile phase. Ten microliters of extracts were injected and eluted at 0.8 mL/min with a linear gradient from 5–80% of solvent B (methanol) in solvent A (Milli-Q water) for 30 min, then an isocratic mode (80% of solvent B) for 10 min, and then a return to the initial conditions in 5 min. The temperature of the column was 20 °C. UV-Vis spectra were recorded at wavelength of 366 nm. Gallic acid, quercetin, (+) catechin standards were used for identification of phenolic compounds in EBM-EtOH25% and EBM-EtOH50% with their retention time used as a reference [40].

### 2.8. Atomic Absorption Spectrometry Analysis of B. macrantha Metal Content

The concentration of 10 metals (Ca, Cu, Fe, Mg, Mn, Zn, Cd, Co, Ni, and Pb) in the leaves of *B. macrantha* were measured:

(a) Ashes: 5 empty crucibles were labeled with graphite pencil, taken to a flask at 550 °C for 4 h, until constant weight. They cooled down in a desiccator for 30 min and their weight was recorded. Then 0.6–0.7 g of flour from the leaves of *B. macrantha* spray was added to each sample. The crucibles with the sample were brought back to the flask at 550 °C for 24 h.

(b) Acid digestion: 1 mL of concentrated HNO_3_, free of trace metals, and 2 mL of fuming HCl, free of trace metals, were added to the ashes contained in the crucibles. The crucibles with the acids were heated to 75 °C for 2 h, to digest the remaining organic material. After, the digestion was completed, the residue from each crucible was filtered with a filter of 0.45 μm and graduated to 25 mL with 1% *v*/*v* HNO_3_. This content was transferred to 150 mL plastic bottles until use.

(c) The measurement of the concentration of metals with the atomic absorption spectrophotometer AA550 (PG Instruments, Leicestershire, UK) and the data were collected through AAWin Pro software, from the same manufacturer. The measurement conditions for metals are shown in Table 1.

### 2.9. Biological Activities

#### 2.9.1. Thiobarbituric Acid Reactive Substances (TBARs) In Vitro Assay

EBM’s capacity to inhibit lipid peroxidation was evaluated by mean of the TBARS assay according to the method described by Carrillo et al. [41] with some modifications. Ten milliliters of olive oil were mixed with EBM or BHT at different concentrations (100, 200, 500, and, 1000 µg/mL). The oil was oxidized by heat at 100 °C for 48 h under continuous agitation. Then oil was cooled and centrifuged at 10,000× *g* for 10 min.

Oxidized oil samples were mixed with TBA (1 mM of TBA in glacial acetic acid 50%) at ratio (1:1 *v*/*v*). Negative control was also prepared using only olive oil. The solution was incubated at 100 °C for 1 h, cooled down for 10 min, and centrifugated. The aqueous phase was measured at 532 nm with a spectrophotometer (Evolution 201, Thermo Scientific, Waltham, MA, USA). The capacity to inhibit lipid peroxidation was expressed as a percentage of inhibition of lipid peroxidation, % Inhibition of lipid peroxidation = (Am-Ac)/(A0-Ac) × 100

Where Am is the absorbance of the sample, Ac is the absorbance of the negative control, and A0 is the absorbance of the olive oil without oxidation.

#### 2.9.2. Red Blood Cell Membrane Stabilizing (RBC) Assay

The evaluation of anti-inflammatory activity by the RBC method was carried out according to the method described by Kota et al. [42] with slight modifications. Blood (5 mL) was collected from a healthy adult volunteer (who was not taking any NSAIDS for two weeks before the experiment) and mixed with an Alsever solution (2% dextrose, 0.8% sodium citrate, 0.5% citric acid and, 0.42% NaCl) in equal quantities. The sample was centrifuged at 2500× *g* for 5 min. The pellet was washed three times with a saline solution (0.85% *w/v*). A 10% *v*/*v* RBC solution was prepared in saline and stored at 4 °C, and 1 mL of EBM at different concentrations (25, 50, 100, 200, 500 and 1000 µg/mL) was mixed with 1 mL of phosphate buffer (0.15 M, pH 7.4), 2 mL of hyposaline solution (0.36%), and 0.5 mL of RBC suspension. The mixture was incubated at 37 °C for 30 min and centrifuged at 1000× *g* for 3 min. The absorbance of the hemoglobin content of the supernatant was measured at 540 nm. The anti-inflammatory drug diclofenac sodium was used as a positive control (25, 50, 100, 200, 500 and 1000 µg/mL) and Milli-Q water was used as a negative control. Each experiment was performed in triplicate. The results were expressed as a percentage of RBC with the following Equation:

% Protection RBC = 100 − (As/Ac × 100)(1)

where As is the absorbance of the sample and Ac is the absorbance of the control.

#### 2.9.3. Evaluation of Antioxidant Activity In Vitro

##### Ferric-Reducing Antioxidant Power (FRAP) Method

The FRAP reagent was prepared by mixing (25 mL of 300 mM sodium acetate buffer at pH 3.60 + 2.50 mL of 10 mM TPTZ diluted in 40 mM HCl + 2.50 mL of 20 mM ferric chloride hexahydrate). Next, 900 µL of FRAP reagent was mixed with 90 µL of distilled deionized water and 30 µL of EBM. The mixture was incubated in the dark at 37 °C for 30 min. It was then centrifuged at 500× *g* for 5 min. Finally, the absorbance of the samples was measured at 593 nm. The trolox standard was used to make a calibration curve (100–500 µmol). The curve obtained was (y = 0.0011x + 0.46, R^2^ = 0.9972). The assay was done in triplicate (*n* = 3). The antioxidant activity data measured by FRAP were expressed as µmol trolox equivalents (TE)/g EBM, dry weight (DW) [43].

##### Azinobis (3-Ethyl-Benzothiazoline-6-Sulfonic Acid) Cation Bleaching ABTS Method

EBM (200 μL) were mixed with 3800 μL of ABTS solution (7 mM ABTS solution + 2.45 mM K_2_S_2_O_8_ in a 1:1, *v*/*v* ratio) and was incubated for 45 min in the darkness. Then, the mix was diluted adding a phosphate buffer (pH 7.0) until an absorbance of 1.10 ± 0.01 at 743 nm was obtained. The Trolox standard solution (0 μmol to 500 μmol) was used as the standard curve to determine the concentrations of antioxidants. The standard curve obtained was (y = 0.0011x + 0.46, R^2^ = 0.9972). The assay was done in triplicate (*n* = 3). The results of the antioxidant activity by ABTS assays were expressed as μmol of trolox equivalents (TE)/g EBM, dry weight (DW) [43].

##### 2,2-Diphenyl-1-Picrylhydrazyl (DPPH) Radical Scavenging Assay

The antioxidant activity of EBM was measured by the DPPH method described by Boeri et al. [44]. The trolox standard was used as the reference standard curve (0–800 µmol Trolox/L) and the calibration curve was obtained (y = 0.0011x + 0.46, R^2^ = 0.9997). All assays were done three times (*n* = 3). The results obtained were represented as µmol trolox equivalents (TE)/g EBM, dry weight (DW).

### 2.10. Statistical Analysis

The results obtained in this study were expressed as the means ± standard deviation of the three experiments (*n* = 3). To determine the statistical differences, the data was evaluated with one-way ANOVA analysis, followed by the Tuckey test. The significant differences were considered at *p* < 0.05.

## 3. Results and Discussion

### 3.1. Effect of Solvent in the Extraction Process of TPC and TFC

The effect of the solvent on the extraction of TPC and TFC was tested using water, ethanol and methanol at a temperature of 50 °C. Table 2 shows the results of the effect of solvent in the extraction of TPC and TFC in EBM.

The highest values of TPC and TFC were achieved using the solvent EtOH50% with values of (12.59 mg GAE/g EBM, DW) and (6.89 CT/g EBM, DW). This solvent was more efficient for the extraction of the phytocomponents than the solvents water and ethanol. The lowest values of TPC and TFC were obtained in the aqueous extracts with values of (6.25 mg GAE/g EBM, DW) and (2.79 mg CT/g EBM, DW), respectively. Ethanol and methanol solvents at high concentrations of 96% and 98% decrease their effectiveness in the extraction of EBM phytocomponents.

According to the statistical analysis made (one way-ANOVA), it is concluded that there are significant differences between the mean TPC and TFC concentrations of the different solvents evaluated. The *p* value of the F test is less than 0.05, for 95% confidence [(F-Ratio 115.38; P-Valor 0.000) to TPC and (F-Ratio 37.54; P-Valor 0.000) to TFC].

Three quality parameters were selected to choose the best solvent in the TPC and TFC extraction. Those parameters were yield, safety, and cost. Under these three parameters, EtOH25% was chosen to optimize the extraction of TFC and EtOH50% for TPC. A wide variety of organic solvents used for secondary metabolite extraction processes such as ethanol, methanol, acetone, propanol, ethyl acetate, dimethylformamide, and their combinations with each other and with water have been reported in the literature.

### 3.2. Effect of Temperature in the Extraction Process of TPC and TFC

Table 3 shows the results of the effect of the temperature in the extraction of TPC and TFC of EBM at 30, 40, 50, and 60 °C.

### 3.3. Effect of Time of Sonication in the Extraction Process of TPC and TFC

Table 4 shows the results of the effect of sonication time on the extraction of TPC and TFC from EBM-EtOH25% and EBM-EtOH50% at 50 °C and 60 °C for 5, 10, and 20 min. The TPC content increases with the sonication time tested. The highest value of TPC at 50 °C was obtained with 5 min of sonication (14.68 mg GAE/g EBM-EtOH50%, DW). The highest TFC value at 50 °C was obtained at 20 min of sonication with a value of (8.75 mg CT/g EBM-EtOH25%, DW). The highest value of TPC at 60 °C was obtained with 20 min of sonication (17.33 mg GAE/g EBM-EtOH50%, DW); the TFC presented a value of (9.84 mg CT/g EBM-EtOH25%, DW). One-way ANOVA analysis of variance showed that temperatures presented statistically significant differences for TPC and TFC. The analysis was evaluated with 95% confidence [(F-Ratio 55.42, *p*-Value 0.000) for TPC and (F-Ratio 124.01, *p*-Value 0.000) for TFC.

Soto et al. [45] have described the ethanolic extract of *B. alnifolia* from Chile (EtOH96%, 30 min of ultrasonic bath) with a content of TPC (4.42 mg GAE/g, DW) and TFC (1.24 mg quercetin equivalents (QE)/g, DW). The reported values of TPC and TFC for the different EBMs were higher than those reported for the ethanolic extract of *B. alnifolia*.

Brighente et al. [46] have described the TPC and TFC content of different extracts of *B. illinita* and *B. platypoda* from Brazil. The extracts were obtained with different solvents during a period of 15 days. The reported contents were (19.91–351.95 mg GAE/g, DW and 5.22 QE/g, DW) for *B. illinita* and (11.29–149.13 mg GAE/g, DW and 7.11–35.87 QE/g, DW) for *B. platypoda*. These TPC and TFC values were higher than those reported in this study for EBM. Differences may be due to the *Baccharis* species, the origin of the crop, the part of the plant used for the extract (leaves, flowers and stems), and the process used to obtain the extracts (solvents, temperatures, time, sonication, supercritical fluids, and number of extractions). Cavalaro et al. [47] have described different ethanolic extracts of *B. dracunculifolia* from Brazil with TPC contents between (11.33–120.52 mg GAE/g, DW). Gomez et al. [48] have described decoction extract of *B. grisebachii* with a content of TPC (62.46 mg GAE/g, extract) and TFC (5.30 QE/g, extract). Sabir et al. [49] have described the TPC and TFC content of water extract of *B. trimera* from Brazil. The extract was concentrated with the help of a rotary evaporator. The values found were (101 mg GAE/g, DW) for TPC and (35.00 mg QE/g, DW) for TFC. Agudelo et al. [50] have reported content of TPC and TFC of methanolic extract of *B. spicata* with values of (66.30 mg tannic acid/g, DW) to TPC and (7.46 mg rutin/g, DW) to TFC.

Once the optimal parameters for the extraction of TPC and TFC in the extracts (solvent, temperature, and sonication time) were established, extractions of TPC and TFC were carried out until the plant material was totally used. TPC and TFC required four extraction cycles to obtain a value of (23.54 mg GAE/g EBM-EtOH50%, DW) and (19.32 mg CT/g EBM-EtOH25%, DW) equivalent to 100% and 98.56%, respectively. TFC needed five extraction cycles to obtain a value of (19.60 mg CT/g EBM-EtOH25%, DW), equivalent to 100%. Consecutive extractions enabled the improved performance of the content of the phytocomponents recovered in the plant material.

**Figure 2:**
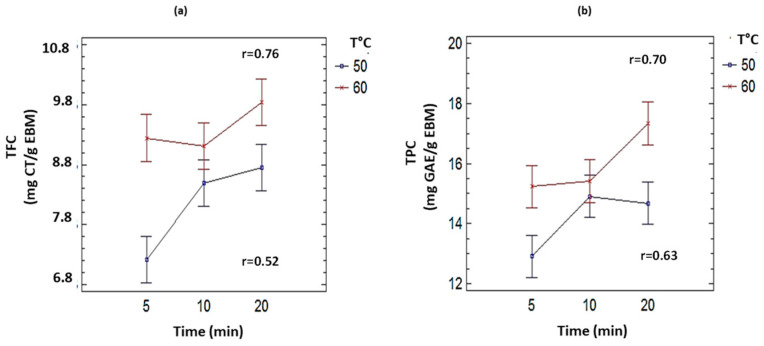
Relationship of temperature and time factors at different levels on (**a**) total flavonoids content (TFC) and (**b**) total polyphenol content (TPC). (r = Pearson coefficient of correlation). (*p* < 0.005).

To determine the effect of the factors (temperature and time) on the response variable (concentration), a factorial design of (A × B) was applied, where factor A (temperature) has two levels (50 °C and 60 °C) and factor B (time) has three levels (5, 10, and 20 min). A multifactorial analysis of variance was performed, with the mean difference for each factor. For the interaction between the different factors, a confidence level of 95% was observed. The correlation between the factors for each of the levels is shown in the interaction graph (Figure 2).

When the intervals for each cell overlap, the effect of the factors (temperature and time) is the same in that cell. The effect is observed in Figure 2a,b for TFC and TPC (50 and 60 °C for 10 min); both temperatures at that time produce the same effect in the polyphenol extraction. Therefore, either of the two temperatures can be chosen as the best treatment. In both cases, it was decided to work with the lowest temperature, or 50 °C for an extraction time of 10 min, to preserve the quality of the extracted metabolites.

A Pearson correlation analysis was performed. It was found that the correlation coefficients were higher when temperature was related to TPC and TFC. Values of r = 0.70 and r = 0.76, respectively, were found. When time was related to TPC and TFC, the coefficients were quite low with values of r = 0.63 and r = 0.52, respectively (Figure 2a,b). This indicates that there is a better correlation between the temperature variable and the concentration variable.

The influence of the sonication time measured from 5 to 20 min on TPC concentration is shown in Table 4. Statistical evaluation (ANOVA) suggests that TPC extraction yields are time dependent. The study shows that the recoveries of phenolic compounds increased with sonication time, reaching the maximum at 10 min of sonication. Extraction efficiencies were low during the first 4 min of sonication, indicating that more sonication time is needed to break down cell walls and release phenolic compounds from cell constituents. The prolonged application of time did not benefit polyphenol yields to a large extent [51]. Since the particle sizes can affect the extraction process and its effectiveness, the experimental work was conducted with the fine powder of dried leaves of *B. macrantha* and the solid–solvent ratio was set to (1:10, *w*/*v*). The ultrasound assisted extraction was used to create optimal conditions, allowing a high diffusion of the solvent across the cell wall plants and the breaking down of the cell walls plants to facilitate the extraction of the compounds of interest present in the solution [52].

### 3.4. Anti-Inflammatory Activity of EBM

The inflammation process is a tissue response to injury, infection or irritation caused by an external agent. During this process, lysosomal enzymes are released that cause damage to macromolecules and cell membranes. The stabilization of lysosomal membranes is important in controlling the inflammation process. RBC is a method based on the stabilization of lysosomal membranes that have been subject to cell lysis due to hypotonicity. The ability of the extracts to stabilize erythrocyte membranes is evaluated with the RBC method [42].

**Figure 3:**
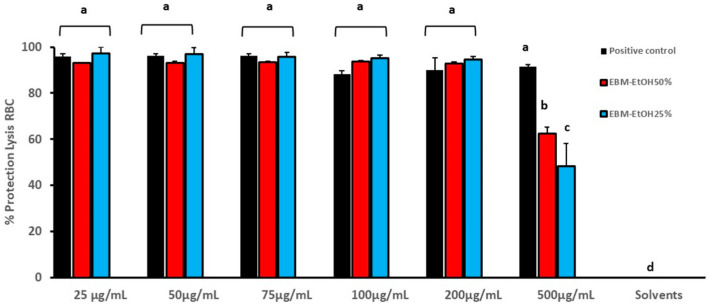
Anti-inflammatory activity of EBM-EtOH25% and EBM-EtOH50% by red blood cell membrane stabilized (RBC) assay. The results for positive control (diclofenac) and negative control solvents (EtOH25% and EtOH50%) can be seen. Results are presented as Mean ± SD (*n* = 3) and were evaluated by one-way Anova and Tukey test (*p* < 0.05). Statistical differences are indicated with different letters.

The anti-inflammatory activity of EBM-EtOH25% and EBM-EtOH50% was evaluated with RBC assay. EBMs were tested at concentrations of 25, 50, 100, 200, and 500 µg/mL. The diclofenac sodium standard was used as positive control. Figure 3 shows results of EBM-EtOH25% and EBM-EtOH50% anti-inflammatory activity by the RBC assay. The positive control (diclofenac) presents a value between (91.42–97.12%) of protection lysis RBC. EBM-EtOH25% and EBM-EtOH50% at 100 and 200 µg/mL present higher values than the positive control. Moreover, 100 µg/mL of EBM-EtOH25% presents a value of 93.70% and EBM-EtOH50% presents a value of 92.26%. EBM-EtOH50% at 25 and 50 µg/mL presents a higher value than the positive control. Only EBM at 500 µg/mL presents statistical differences of the samples compared to the positive control after analysis of one-way ANOVA at *p* < 0.05. But EMB-EtOH25% and EBM-EtOH50% at 500 µg/mL were less active than the positive control.

De Oliveira et al. [53] have described six extracts of *B. trimera* with anti-inflammatory activity, using carrageenan-induced pleurisy in a rat model. The extracts were tested at concentrations of (25, 50, and 75 mg/kg). All the tested extracts showed anti-inflammatory activity. In addition, the extracts were enriched with phenolic compounds. Observing that the most active extract presented a 22.34% inhibition compared to the control carrageenan. Nogueira et al. [54] observed extracts and fractions of *B. trimera* with anti-inflammatory activity for carrageenan-induced mouse paw edema. Treatment with AFBt (aqueous fraction of *B. trimera*) reduced paw edema at 3 h by 44.70–54.20% and EFBt (ethanolic fraction of *B. trimera*) reduced edema by 49.30–58.20% compared to positive control (indomethacin 10 mg/kg), which reduced 64.60% of edema at 3 h. Zalewski et al. [55] described ethanolic extracts of *B. uncinella* with strong anti-inflammatory activity against inflammatory reactions induced by phospholipase A2 and rat paw edema induced by carrageenan model.

Different *Baccharis* species have shown anti-inflammatory activity in different in vivo and in vitro evaluation models. The anti-inflammatory activity of *B. macrantha* has not been reported in the scientific literature.

### 3.5. Inhibition of Lipid Peroxidation of EBM

**Figure 4:**
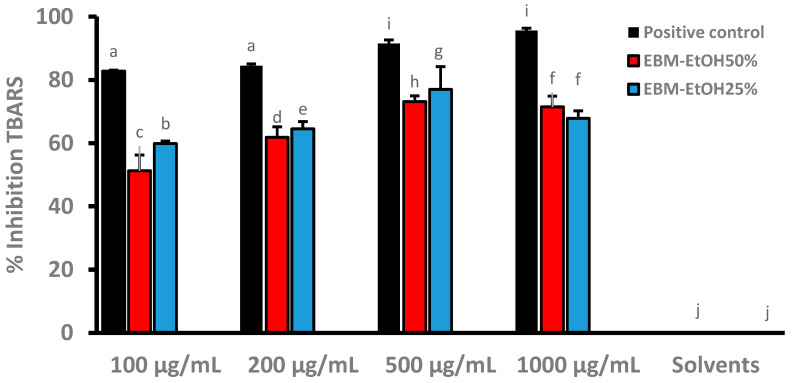
Inhibition of lipid peroxidation in vitro of EBM-EtOH25% and EBM-EtOH50%. Results for positive control butylhydroxytoluene (BHT) and negative control solvents (EtOH25% and EtOH50%) can be seen, as well as the results for EBM (extracts of *Baccharis macrantha*). Results are presented as Mean ± SD (*n* = 3) and were evaluated by one-way Anova and Tukey test (*p* < 0.05). Statistical differences are indicated with different letters.

Figure 4 shows the results of EBM-EtOH25% and EBM-EtOH50% inhibition of lipid peroxidation in vitro. The samples were tested at concentrations of 100, 200, 500, and 100 µg/mL of EBMs. BHT standard was used as positive control. When the samples were compared to the positive control, they showed less activity than the positive control at all concentrations tested. At 500 µg/mL of BHT, the sample presented a value of 91.42%. The other samples showed a value of 77.00% in EBM-EtOH25% and 73.11% in EBM-EtOH50%. De las Heras et al. [56] have described ethanolic extracts of *B. trinervis* at a concentration of (100 µg/mL) with inhibition of microsomal lipid peroxidation of 74.80% of inhibition compared with control BHT (100 µM) with 100% of inhibition. Tapia et al. [57] reported extracts of *B. grisebachii* at a concentration of (500 µg/mL) with a capacity to inhibit lipid peroxidation in erythrocytes with values between 32–58% of inhibition. Our %TBARS inhibition values were higher than those reported in the previous study. Sabir et al. [49] have described the aqueous extract of *B. trimera* able to inhibit TBARS molecules, induced by different prooxidants (iron and sodium nitroprusside) in rat liver, brain, and phospholipid homogenates from egg yolk. Guimarães et al. [58] described glycolic extracts from *B. dracunculifolia* (GEBd) with capacity to inhibit lipid peroxidation in rat liver mitochondria. GEBd 0.03% presented a value of 75% of inhibition of TBARS molecules. The percentage of inhibition of lipid peroxidation values of EBMs are consistent with those reported in the literature for extracts from other *Baccharis* species.

### 3.6. Antioxidant Activity of EBM

Table 5 shows the results of antioxidant activity of EBM-EtOH25% and EBM-EtOH50% by ABTS, FRAP, and DPPH methods. EBM-EtOH25% and EBM-EtOH50% presented similar antioxidant values when evaluated with the ABTS method (1172 µmol TE/g, EBM and 1168 µmol TE/g, EBM), respectively. Statistical analysis shows that there are no statistical differences between both values. The activity evaluated with the FRAP and DPPH methods present statistical differences. Gallic acid, quercetin, and catechin standards were used as positive controls. The antioxidant activity values evaluated with the three methods, and the results were higher than the two tested extracts (Table 5).

The antioxidant activity of EBM-EtOH25% evaluated by the DPPH method shows an activity 2.20 times higher than the activity of EBM-EtOH50% evaluated with the same method. Parejo et al. [59] described the antioxidant activity of extracts of *B. pentlandiiand* and *B. platypoda*, using the DPPH method. Obtaining values of (IC_50_ = 15.0 to 63.8 µg/mL) and (IC_50_ = 20.5 to 80.9 µg/mL), respectively. Saber et al. [49] have described the antioxidant activity by the DPPH method of the aqueous extract of *B. trimera* from Brazil. The water extract showed values between 30% and 80% DPPH. Soto et al. [45] examined the antioxidant activity of the ethanolic extract of *B. alnifolia* from Chile, and they identified the activity through the FRAP and TEAC methods. They found a value of (13.29 µmol TE/g, DW) through the FRAP method and (6.19 µmol TE/g, DW) through the TEAC method. The FRAP value of EBM was higher than that of the *B. alnifolia* extract.

Gomez et al. [48] have described *B. grisebachii* decoction extract with antioxidant activity evaluated by FRAP and TEAC methods. The extracts showed FRAP (700 µM TE/g extract) and TEAC (0.61 mg TE/g extract) values. The antioxidant values reported in this study for EBM with the FRAP method were higher than those reported in the previous study.

### 3.7. Characterization of EBM by TLC and UHPLC Analysis

**Figure 5:**
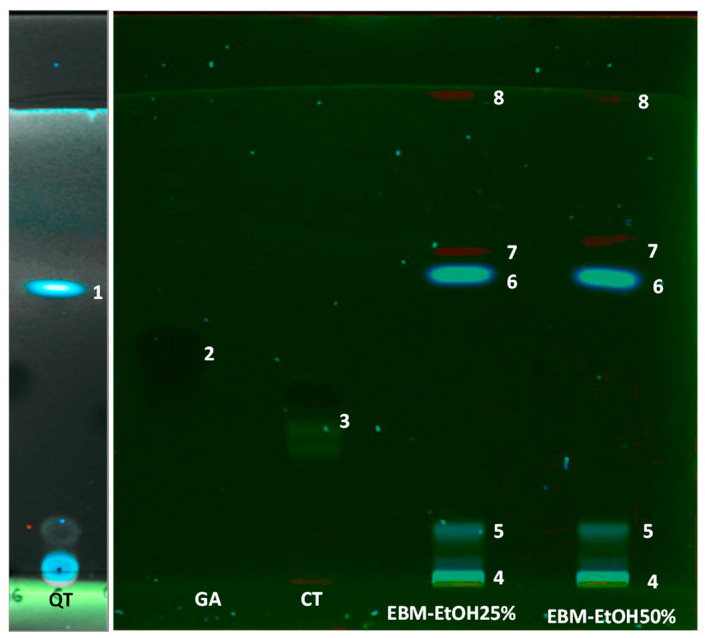
Analysis TLC of EBM-EtOH25% and EBM-EtOH50%. GA (gallic acid standard), CT (+ catechin standard) and QT (quercetin standard). UV light at 366 nm.

The components of EBM-EtOH25% and EBM-EtOH50% were analyzed by the TLC technique (Figure 5). Gallic acid standard (GA), quercetin standard (QT), and catechin standard (CT) were used for the analysis. Figure 5 shows the EBM-EtOH25% and EBM-EtOH50% polyphenol components profile. The bands named 1, 2, and 3 correspond to the standards quercetin, gallic acid, and catechin, respectively. Five bands named 4, 5, 6, 7, and 8 were found in both samples. The bands 4, 5, and 6 were of blue color intensity. The bands 7 and 8 were of red color with low intensity. The intensity of the bands indicates the concentration of the component. The bands 4 and 5 may be associated with methylated or glycosylated phenolic compounds; the bands 7 and 8 would correspond to compounds with hydroxyl or methoxy substituents. The retention factor (Rf) of EBMs has been calculated at this value to describe the polarity of the components. The bands with low Rf correspond to components with high polarity. High Rf is considered components of low polarity. In terms of polarity, GA has the highest polarity with a value of Rf (0.38), followed by CT with a Rf value of (0.42). QT (band 1) was the standard with low polarity with a value of Rf (0.62).

The bands of blue color intensity band 6 (EBM-EtOH25% and EBM-EtOH50%) present values of Rf of 0.61, and the QT (band 1) standard presents an Rf of 0.62. Bands with the same color and the same Rf were identified as the same molecule (quercetin). Bands 2 and 3, which correspond to standard gallic acid and quercetin, respectively, were not detected in the two extracts tested. QT is a hydroxy flavone with 3 phenolic rings and 5 hydroxyl groups. The bands 4, 6, 7, and 8 observed in both EBM samples were not identified.

Different Chinese medicinal plants are used to treat and prevent neurodegenerative diseases such as Alzheimer’s and Parkinson’s. Different flavonoid compounds have been identified from medicinal plant extracts such as apigenin (4′,5,7-trihydroxyflavone), luteolin (7-O-β-glucoside), and quercetin. It has been described that flavonoids are compounds with the ability to prevent neurodegenerative diseases caused by dietary intake of advanced glycation end products from modern diets. Yang et al. [60] reported quercetin with preventive effects against cognitive damage of mice produced by advanced glycation end-products in the diet. Many food plants have quercetin flavonoids and are reported to have protective effects against Alzheimer disease in human patients and mouse models [61]. Oyedemi et al. [62] reported quercetin with the capacity to reduce hyperglycemia in a type 2 diabetes rat model.

**Figure 6:**
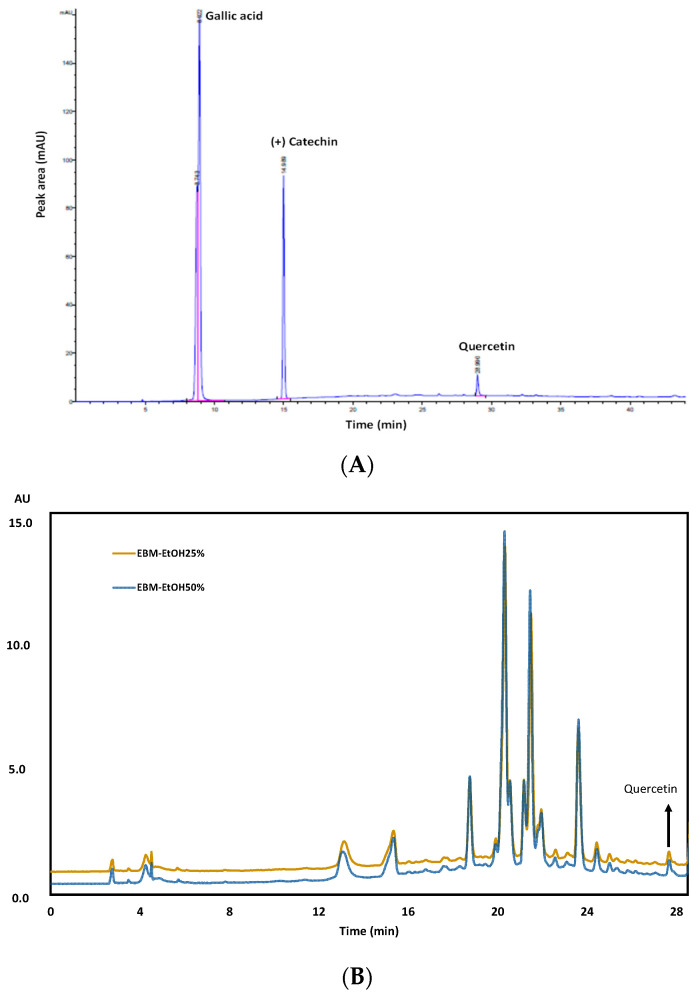
Analysis UHPLC of extracts of *Baccharis macrantha*. (**A**) Gallic acid, catechin, and quercetin standards (**B**) EBM-EtOH25% and EBM-EtOH50%. (λ = 366 nm).

The components of EBM-EtOH25% and EBM-EtOH50% were also characterized with the UHPLC technique at a wavelength of 366 nm (Figure 6). GA, CT, and QT standards were used as reference in the analysis at concentration of 100 µg/mL (Figure 6A). According to the retention times of the standards, only QT was identified in the chromatogram at wavelength of 366 nm of EBM-EtOH25% and EBM-EtOH50%) at retention time of 28.98 min (Figure 6B). This result is in accordance with the results obtained in the TLC analysis of EMB-EtOH25% and EBM-EtOH50%. The chromatogram presents a complex profile with compounds that were not identified by UHPLC between retention times of 2 to 26 min.

Simões-Pires et al. [63] have identified polyphenolic and flavonoid compounds in aqueous extracts of *B. trimera*, *B. crispa*, and *B. usterii*. The main compound identified in all three species was quinic acids esters. Isoquercetin and quercetin compounds were identified in *B. trimera*. These two flavonoids were detected by HPLC-MS as the abundant compounds in *B. trimera*. Moreira et al. [21] have identified flavonoid compounds in methanolic extracts of *B. pseudotenuifolia*, using the TLC technique. Among the identified compounds, quercetin, 3′-methoxy-quercetin, quercetin-3-O-rhamnoside and, quercetin-3-O-glucoside were found.

Different flavonoid compounds have been described in *Baccharis* species. The quercetin compound has been identified in species such as *B. grisebachii*, *B. latifolia*, *B. illinita,* and *B. pseudotenuifolia* [19]. Saber et al. [49] have identified and quantified gallic acid (45.8 ± 7.2 mg/g), rutin (15.1 ± 2.5 mg/g), and quercetin (5.1 ± 1.4 mg/g) in extracts of *B. trimera*; the identification was made by HPLC technique. Wollenweber et al. [64] have identified flavonoid compounds from extracts of *B. concinna*, *B. confertifolia*, *B. linearis*, and *B. lycioides*. Within these compounds, they identified quercetin, quercetin 3-Me, quercetin 7-Me, and other isoforms. The differences in the identifications of the compounds may be due to the species of *Baccharis*, the way of obtaining the extracts, and the type of analysis used for the identification.

### 3.8. Quantification of Mineral and Heavy Metals Content in B. macrantha Leaves

Dry leaves of *B. macrantha* were used to determine the cadmium (Cd), calcium (Ca), cobalt (Co), cupper (Cu), iron (Fe), magnesium (Mg), manganese (Mn), Nickel (Ni), Lead (Pb), and Zinc (Zn) concentrations recorded by the atomic absorption spectrophotometer. Table 6 shows the content of minerals and heavy metals in dry *B. macrantha* leaves.

Cd concentrations in contaminated plants and soils have been reported, and these reports are based on the regulations of the USA Environmental Protection Agency (EPA), which indicate that the maximum concentration of Cd in uncontaminated conditions is equal to 0.20 mg/kg of plant [65]. However, the normal level must be equal to or less than 0.10 mg/kg of plant. As observed in Table 6, such values indicate that there is no Cd contamination in the *B. macrantha* plants, especially by anthropogenic sources. The values obtained are within the normal range accepted by the EPA.

The Co concentration varies depending on the type of soil. However, values between 1 to 40 ppm of Co are considered normal. Of the total existing Co in the soil, a small part is bioavailable for plants; the distribution of (Co) in plants depends on the species. Wild forage plants of different species have been found with a Co content between 0.6 and 3.5 ppm, while other edible plants such as lettuce and cabbage have contents above 0.6 ppm [66]. The values are considered normal for plants; *B. macrantha* has enough Co content to fulfill its metabolic functions, and there is no contamination of this metal by geogenic sources, such as the ash of the Tungurahua volcano. Ni content in *B. macrantha* is below the limit of quantification of the atomic absorption equipment. The value reported for Ni, is lower than 0.238 mg Ni/kg, DW. Chen et al. [67,68] assures that the Ni concentration in most plants is very low, between 0.05 to 10 mg Ni/kg, DW. The content of Mg, Mn, Zn, Ca, and Cu is in accordance with the concentration reported in the literature on plants.

Heavy metal concentrations are generally low in plants because the leaves of *B. macrantha* were collected in the cloud forest “Teligote”, a forest with generally little human activity, and generally, its concentration is of Geogenic origin or from volcanic eruptions or leaching from mineralization of the bedrock [68].

Deterioration of food during different stages of the production line (processing, distribution, and storage) can be caused by chemical and microbial treatments. Oxidative deterioration involves mechanisms of peroxidation of unsaturated fats often phospholipids through a series of free-radical chain reactions. The formation and decomposition of hydroperoxides give place to oxidized compounds of low molecular weight such as ketones, aldehydes, alkanes, and alkenes determining the sensorial quality of food products [69]. This negative effect can be reduced using concentrations of synthetic antioxidants (BHT and BHA), which are effective, stable, and low cost. However, the synthetic antioxidants can be related to a possible mutagenicity and cell damages [70,71]. Natural alternatives with a capacity to inhibit lipid peroxidation have gained importance in scientific and technological fields. Plant extracts can be used as natural agents in preservation of foods since their potential as antioxidant products can replace synthetic antioxidants. These are natural alternatives without secondary effects in health or in sensory aspects of foods. Important uses can be found in strategic fields such as the pharmacological cosmetic or food industries [72].

The interest in preventive medicine that use “natural products” from plant materials has increased worldwide. Many studies describe biological effects such as anti-inflammatory, antimicrobial, and antioxidant activities [73] of compounds isolated from plant tissues. These biological systems synthetize a wide variety of compounds such as phenolic compounds, carotenoids, vitamins, among others with different effects [74].

Different works suggest that a diet rich in foods with a high content of bioactive compounds such as cereals, legumes, nuts, olive oil, vegetables, fruits, tea, red wine, among others, can have a protective and preventive effect on different cancers, cardiovascular diseases, coronary heart diseases, neurodegenerative diseases, inflammation processes, and damage caused by cellular aging. Furthermore, the protective associations between phenolics compounds and these neurodegenerative diseases have also been described [75].

## 4. Conclusions

*B. macrantha* is a plant with medicinal properties used to treat different inflammatory diseases in Ecuador. Plant extracts were obtained from leaves of *B. macrantha* grown in volcanic areas of Ecuador. The extracts of *B. macrantha* presented high values of TPC and TFC. The most suitable solvent to obtain a high content of phenolic compounds was EtOH25% and EtOH50%.

EBM-EtOH25% and EBM-EtOH50% present in vitro high anti-inflammatory and antioxidant activities. EBM showed the ability to inhibit lipid peroxidation, and this ability was observed when the in vitro method was used. In both extracts, the quercetin flavonoid molecule was identified by TLC and UHPLC techniques. Future studies can focus on the identification of other phenol compounds present in EBM, using the mass spectrometry technique. The extracts can be subject to gastrointestinal digestion simulation to determine the enzymatic hydrolysis of phenolic compounds. EBM has an interest for future studies for the medical and pharmaceutical industries for its biological properties.

## Figures and Tables

**Table 1:** Conditions of measure of metals in dry leaves of *B. macrantha*.

Metal	Method	Wavelength (nm)	Calibration Curve (ppm)	Lamp Current (mA)	Type of Flame
Copper	Flame	324.79	0.1–1.75	5.0	Air/Acetylene
Iron	Flame	248.30	0.3–3.00	5.0	Air/Acetylene
Magnesium	Flame	285.20	0.04–0.40	5.0	Air/Acetylene
Manganese	Flame	279.50	0.2–1.40	5.0	Air/Acetylene
Zinc	Flame	213.90	0.1–2.00	5.0	Air/Acetylene
Calcium	Flame	422.70	0.5–3.50	5.0	N_2_O/Acetylene
Cobalt	Graphite furnace	240.70	0.015–0.10	7.0	10
Nickel	Graphite furnace	232.0	0.0375–0.30	4.0	10
Lead	Graphite furnace	283.30	0.05–0.60	3.0	10
Cadmium	Graphite furnace	228.80	0.002–0.06	5.0	10

**Table 2:** Effect of solvent in the extraction of total polyphenols content (TPC) and total flavonoids content (TFC) of extracts of *B. macrantha* obtained at 50 °C.

Solvent (%)	TPC (mg GAE/g EBM, DW)	TFC (mg CT/g EBM, DW)
Water	6.25 ± 0.44 ^b^	2.79 ± 0.02 ^a^
EtOH-25%	11.05 ± 0.42 ^d^	7.42 ± 0.25 ^e^
EtOH-50%	12.59 ± 0.23 ^e^	6.89 ± 1.05 ^d^
EtOH-75%	10.54 ± 0.70 ^d^	6.29 ± 0.23 ^d^
EtOH-96%	7.90 ± 0.38 ^c^	2.98 ± 0.37 ^a^
MetOH-25%	7.45 ± 0.16 ^c^	6.48 ± 0.83 ^d^
MetOH-50%	7.05 ± 0.34 ^c^	5.51 ± 0.53 ^c^
MetOH-75%	10.82 ± 0.76 ^d^	7.37 ± 0.12 ^e^
MetOH-98%	3.94 ± 0.14 ^a^	4.11 ± 0.20 ^b^

Results are presented as Mean ± SD (*n* = 3) and were evaluated by one-way Anova and Tukey test (*p* < 0.05). Statistical differences are indicated with different letters. DW (dry weight), GAE (gallic acid equivalents), EBM (extract of *Baccharis macrantha*), CT (catechin).

**Table 3:** Effect of the temperature in the extraction of total polyphenol content (TPC) and total flavonoids content (TFC) of EBM-EtOH50% and EBM-EtOH25%.

Temperature (°C)	TPC (mg GAE/g EBM-EtOH50%, DW)	TFC (mg CT/g EBM-EtOH25%, DW)
30	10.83 ± 0.16 ^a^	5.01 ± 0.38 ^a^
40	10.82 ± 0.31 ^a^	7.18 ± 0.19 ^b^
50	12.91 ± 0.77 ^b^	7.22 ± 0.23 ^b^
60	15.23 ± 0.49 ^c^	9.24 ± 0.24 ^c^

Results are presented as Mean ± SD (*n* = 3) and were evaluated by one-way Anova and Tukey test (*p* < 0.05). Statistical differences are indicated with different letters. DW (dry weight), GAE (gallic acid equivalents), EBM (extract of *Baccharis macrantha*), CT (catechin).

**Table 4:** Effect of the time of extraction of total polyphenols content (TPC) and total flavonoids content (TFC) of EBM-EtOH50% and EBM-EtOH25%.

Temperature (°C)	Time (min)	TPC (mg GAE/g EBM-EtOH50%, DW)	TFC (mg CT/g EBM-EtOH25%, DW)
50	5	12.91 ± 0.77 ^a^	7.22 ± 0.23 ^a^
	10	14.68 ± 0.30 ^b^	8.50 ± 0.49 ^b^
	20	14.91 ± 0.63 ^b^	8.75 ± 0.25 ^b^
60	5	15.23 ± 0.49 ^c^	9.24 ± 0.24 ^c^
	10	15.41 ± 0.36 ^c^	9.11 ± 0.13 ^c^
	20	17.33 ± 0.40 ^d^	9.84 ± 0.23 ^c^

Results are presented as Mean ± SD (*n* = 3) and were evaluated by one-way Anova and Tukey test (*p* < 0.05). Statistical differences are indicated with different letters. DW (dry weight), GAE (gallic acid equivalents), EBM (extract of *Baccharis macrantha*), CT (catechin).

**Table 5:** Antioxidant activity of extract *Baccharis macrantha* (EBM) by ABTS, FRAP and DPPH methods.

Sample	ABTS (µmol TE/g, EBM)	FRAP (µmol TE/g, EBM)	DPPH (µmol TE/g, EBM)
EBM-EtOH25%	1172 ± 16.77 ^a^	836 ± 37.10 ^a^	85.70 ± 17.70 ^b^
EBM-EtOH50%	1168 ± 16.70 ^a^	930 ± 36.98 ^b^	39.03 ± 17.17 ^a^
Gallic acid	40,000 ± 1790 ^d^	4541 ± 51.48 ^e^	7376 ± 360 ^e^
Catechin	27,142 ± 645 ^b^	1022 ± 40.44 ^c^	4205 ± 111 ^c^
Quercetin	37,628 ± 711 ^c^	3471 ± 51.48 ^d^	6419 ± 284 ^d^

Results are presented as Mean ± SD (*n* = 3) and were evaluated by one-way Anova and Tukey test (*p* < 0.05). Statistical differences are indicated with different letters: GAE (gallic acid equivalents), EBM (extract of *Baccharis macrantha*), TE (trolox equivalents).

**Table 6:** Concentration of metals in dry leaves from *B. macrantha*.

Cation	mg Cation/kg, DW
Cadmium	0.09 ± 0.5
Calcium	8780.83 ± 812.88
Cobalt	0.52 ± 0.03
Cupper	8.59 ± 1.98
Iron	59.71 ± 2.68
Magnesium	2530.30 ± 386.62
Manganese	292.25 ± 18.41
Nickel	<0.24
Lead	<0.17
Zinc	37.78 ± 14.27

Results were expressed as Mean ± standard deviation (*n* = 3). DW (dry weight).

## Data Availability

Not applicable.
